# Deciphering the Neuropsychiatric Lupus Enigma: Navigating the Intersection of Acute Inflammatory Demyelinating Polyneuropathy (AIDP), Infection, and Flare

**DOI:** 10.7759/cureus.53156

**Published:** 2024-01-29

**Authors:** Jhasaketan Meher, Jivtesh Singh, Saroj Kumar Pati, Vinay R Pandit, Saurabh K Patel, Mallika Kapur, M S Nithin

**Affiliations:** 1 Internal Medicine, All India Institute of Medical Sciences, Raipur, Raipur, IND; 2 Radiodiagnosis, All India Institute of Medical Sciences, Raipur, Raipur, IND

**Keywords:** pulse steroid therapy, cyclophosphamide, mrsa, sle, neuro lupus, aidp

## Abstract

A 17-year-old male diagnosed with systemic lupus erythematosus (SLE), showing poor compliance with medication, presented to our facility with a 20-day history of fever, polyarthritis, and cough. Additionally, he had experienced a seizure episode, followed by a one-day history of altered mentation. Subsequently, he developed pneumonia, respiratory distress, and shock, necessitating ventilator and inotropic support. Neuropsychiatric lupus (NP-lupus) was suspected, and hence high-dose steroids, hydroxychloroquine, and broad-spectrum antibiotics were initiated. Following successful extubation, he manifested ascending flaccid paralysis. The presence of albumin-cytological dissociation and axonal neuropathy confirmed the diagnosis of acute inflammatory demyelinating polyneuropathy (AIDP). He underwent further management with pulse steroids and plasmapheresis. Upon recovery, he was discharged on a regimen of steroids, cyclophosphamide, and hydroxychloroquine. During follow-up, he maintained ambulatory status with no residual neurological sequelae.

## Introduction

Systemic lupus erythematosus (SLE) is an extremely heterogeneous clinical entity, exhibiting a wide range of presentations from simple skin and joint involvement to complex, rare manifestations such as neuropsychiatric lupus (NP-lupus). Although we have known about the neuropsychiatric manifestations of lupus for nearly 150 years, initially described by Kaposi, they remain poorly understood in terms of diagnostic accuracy where it remains mostly a diagnosis of exclusion and its management where the treatment guidelines remain unclear [1]. Peripheral nervous system (PNS) involvement is even more infrequently encountered compared to central nervous system (CNS) involvement [2]. Additionally, when compounded by infections, distinguishing between infection and flare itself becomes very challenging.

## Case presentation

A 17-year-old male presented to us with a history of fever, joint pain, and cough for 20 days and headache and vomiting for three days; he had also experienced an episode of seizure and complained of altered mental status for one day. He had been diagnosed with SLE with acute typical cutaneous lesions five months ago but was non-compliant with medication and had been lost to follow-up.

**Figure 1:**
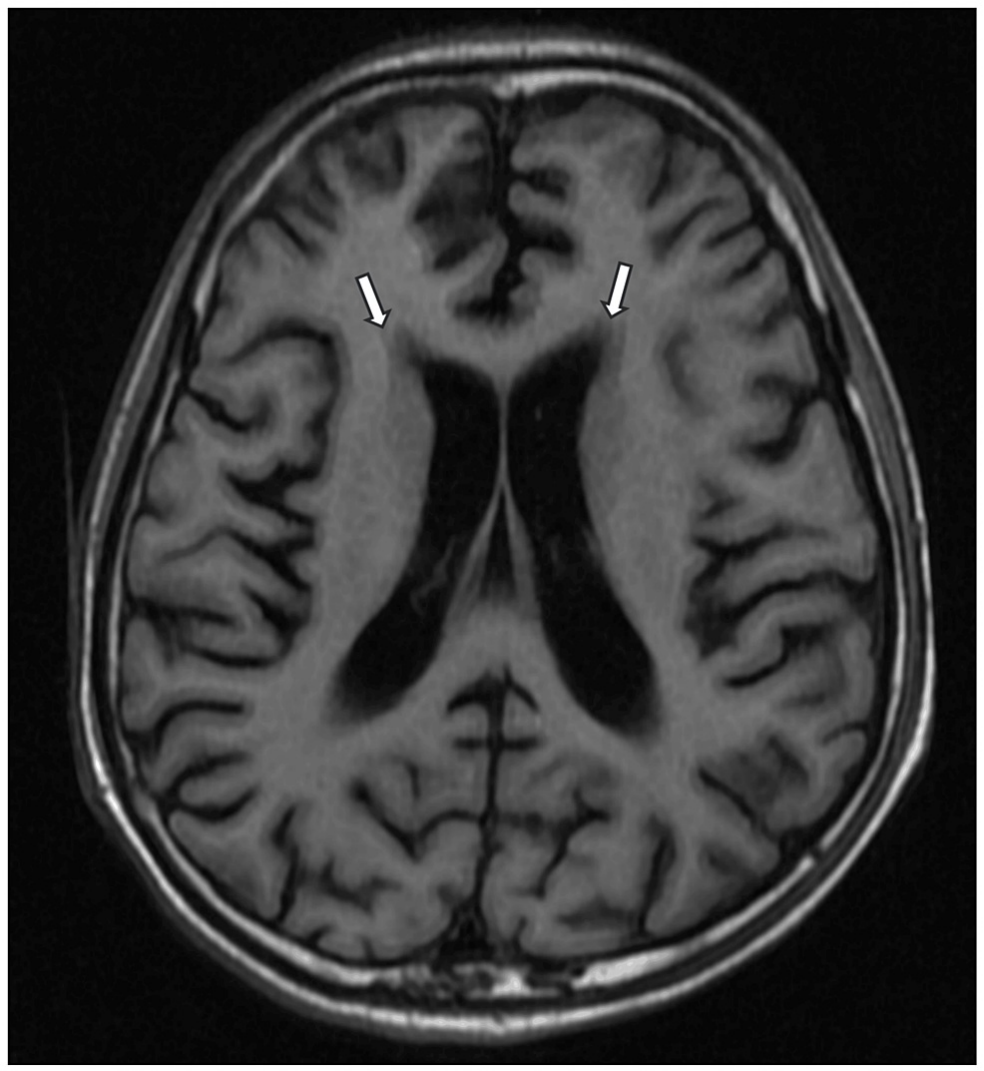
T1-weighted MRI brain (axial section) reveals diffuse periventricular white matter hypointensities (white arrow) MRI: magnetic resonance imaging

**Figure 2:**
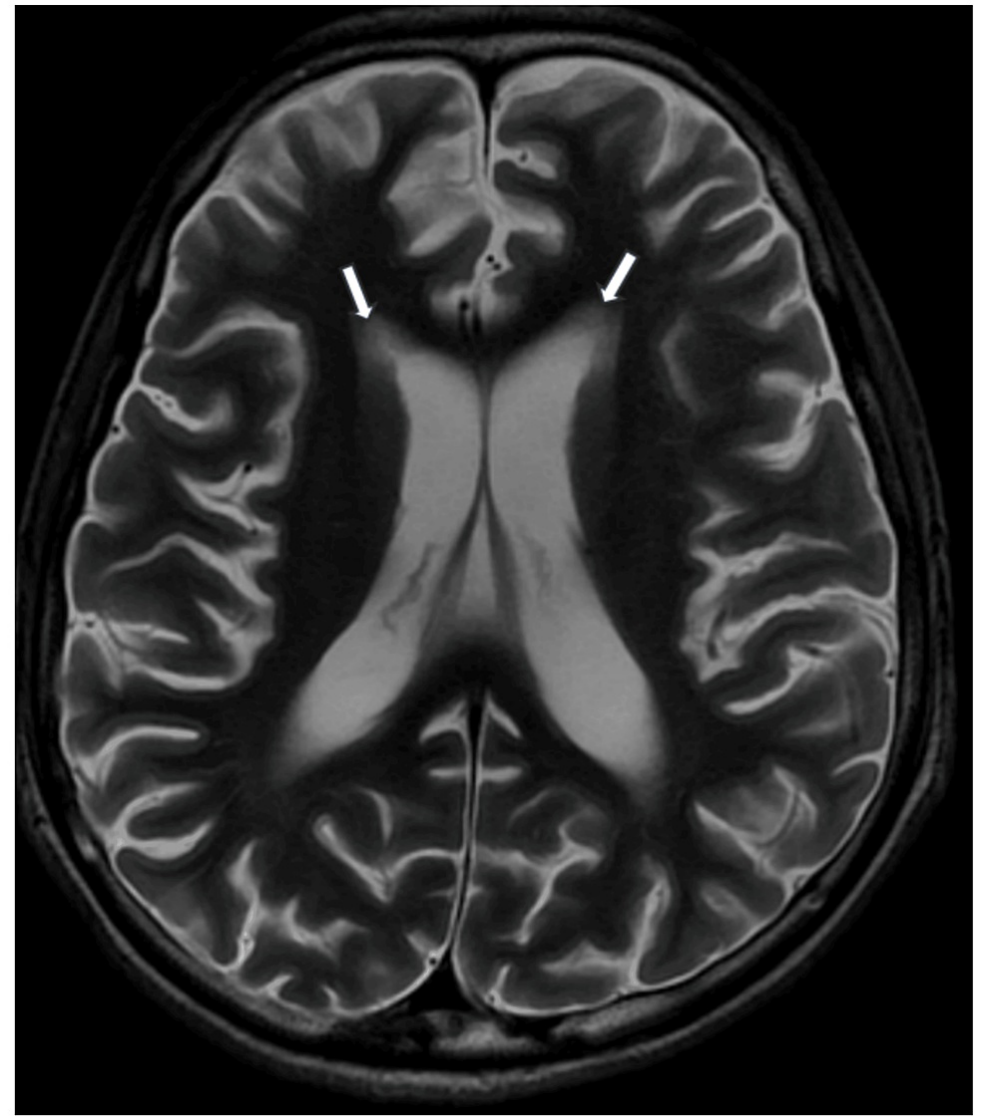
T2-weighted MRI brain (axial section) reveals diffuse periventricular white matter hyperintensities (white arrow) MRI: magnetic resonance imaging

**Figure 3:**
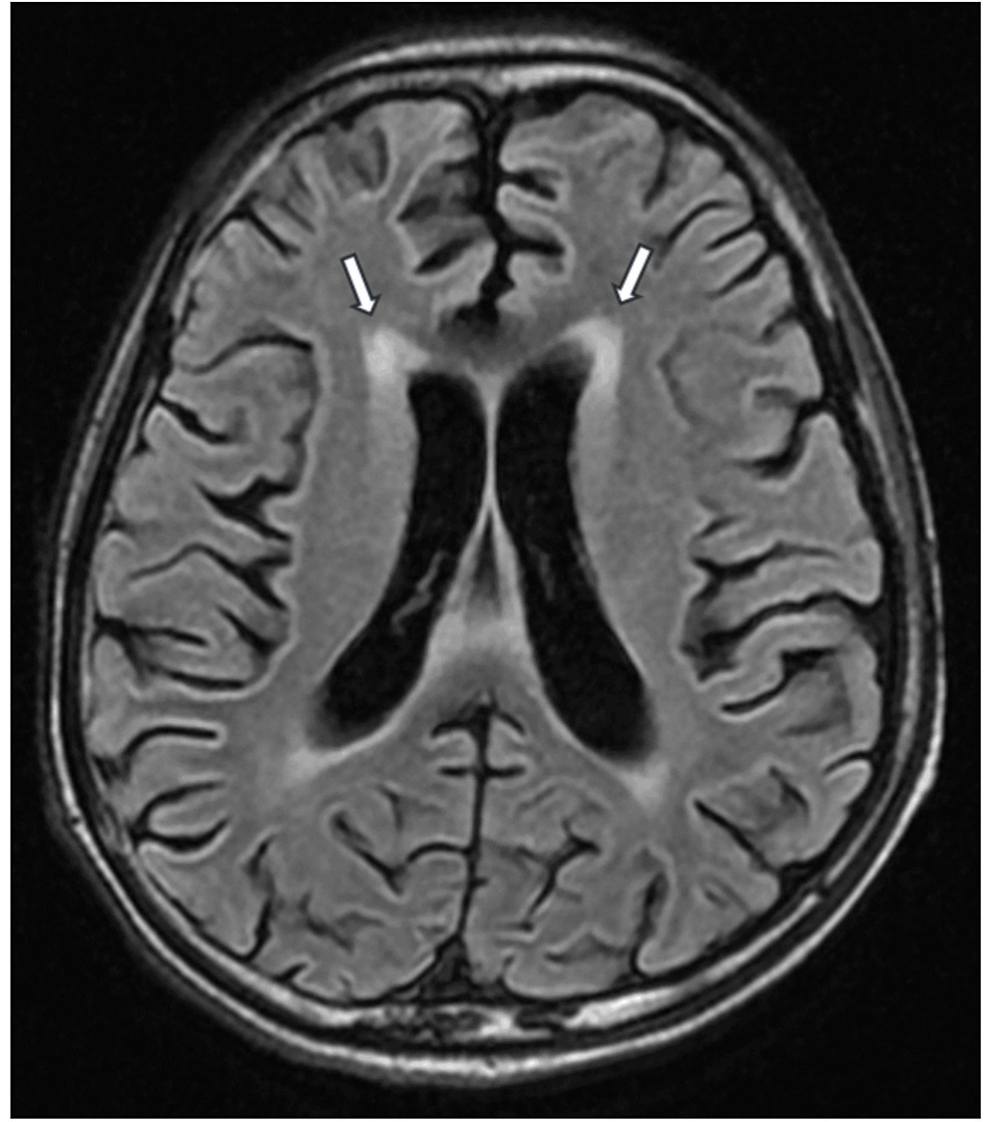
Flair MRI brain (axial section) reveals diffuse periventricular white matter hyperintensities (white arrow) MRI: magnetic resonance imaging

**Figure 4:**
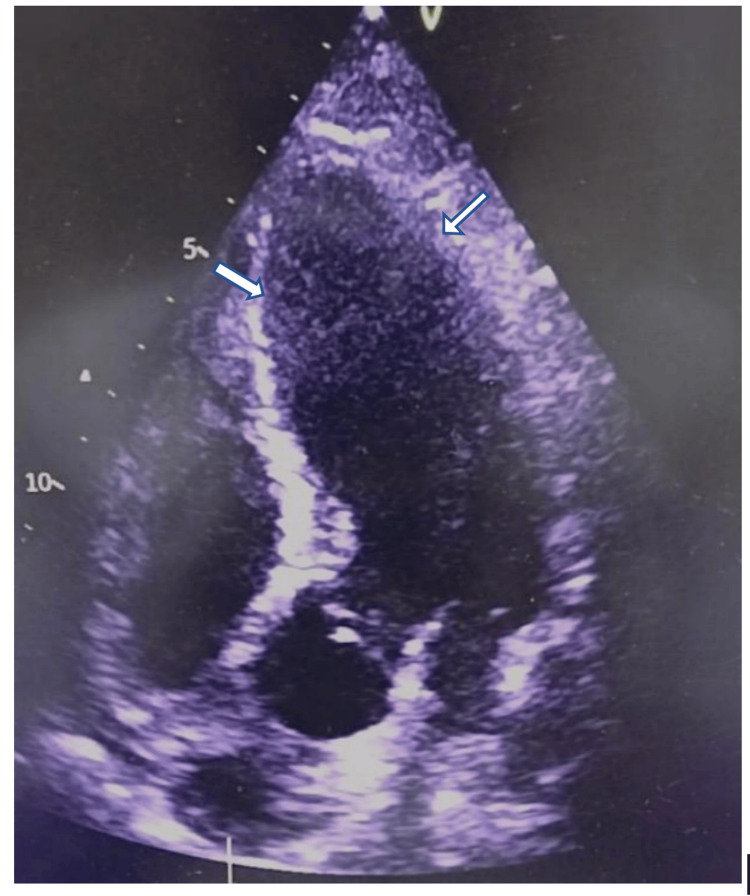
Echocardiography of the heart (apical view) shows a mildly dilated left ventricle with an ejection fraction of 20-25% (white arrow)

The patient presented with an SLE flare-up with pancytopenia (Table 1) complicated by pneumonia. Blood investigations showed high C-reactive protein levels, high erythrocyte sedimentation rate (Table 1), normal liver and renal function, positive anti-nuclear antibody, anti-dsDNA, low C3, and low C4 (Table 2). MRI of the brain revealed ischemic changes in bilateral periventricular areas (Figures 1-3). The management was initiated with broad-spectrum antibiotics, high-dose steroids, and other supportive treatments. The patient continued to deteriorate, developing respiratory distress, hypotension, and hypoxemia, requiring ventilatory and inotropic support. Echocardiography showed global hypokinesia (left ventricular ejection fraction: 20-25%) (Figure 4). Blood cultures revealed methicillin-resistant Staphylococcus aureus (MRSA), which was managed with vancomycin. Bronchoalveolar lavage samples grew multidrug-resistant (MDR) Klebsiella, which was treated with cefoperazone-sulbactam and levofloxacin. Multiple blood transfusions were administered for persistent anemia. He was gradually weaned off from the ventilator and successfully extubated.

**Table 1:** Basic laboratory investigation results of the patient CBC: complete blood count; Hb: hemoglobin; TLC: total leukocyte count; DLC: differential leukocyte count; PLT: platelet count; MCV: mean corpuscular volume; MCH: mean corpuscular hemoglobin; MCHC: mean corpuscular hemoglobin level; RDW: red-cell distribution width; ESR: erythrocyte sedimentation rate; CRP: C-reactive protein; RFT/KFT: renal function test/kidney function test; LFT: liver function test; TB/DB: total/direct bilirubin; SGOT: serum glutamic-oxaloacetic transaminase; SGPT: serum glutamic pyruvic transaminase; TP/Alb: total protein/albumin; GGT: gamma-glutamyl transferase; ALP: alkaline phosphatase; PT/INR: prothrombin time/international normalized ratio

Investigation	Patient value on day 1	Patient value on day 15	Reference range
CBC			
Hb	6.4	6.8	4.5–6.5 x 10^12^/L
TLC	1.96	2.8	4.00–11.00 x 10^9^/L
DLC (N/L/M/E)	75/16.3/8.2/0	69/20/10/1	N: 40–60%, L: 20–40%, M: 2–8%, E: 1–4%
PLT	75	98	150–400 x 10^9^/L
MCV	85.7	89	78–100 fL
MCH	25.3	24.8	27.0–32.0 pg
MCHC	295	290	310–370 g/L
RDW	21.7	21.8	11.5–15.0
ESR	30		<20 mm/hr
CRP	86.6	26.11	<0.3 mg/dL
RFT/KFT			
Urea	30	27	<30 mg/dL
Creatinine	0.36	0.37	0.7–1.3 mg/dL
Sodium	140	139	135–145 mEq
Potassium	4.01	4.14	3.5–5.5 mEq
LFT			
TB/DB	0.57/0.20	0.42/0.13	TB: 0.2–1.3mg/dL/DB: <0.3 mg/dL
SGOT/SGPT	48/62	13/18	SGOT: 5–40 IU/L/SGPT: 5–40 IU/L
TP/Alb	5/2.9	5/3.1	TP: 5.5–8.5 g/dL/Alb: 3.5–5.5 g/dL
GGT	70	35	0–25 IU/L
ALP	167	149	20–150 IU/L
PT/INR	11.6/1.1	10.8/1.1	PT: 10–14/INR: 1.0–1.4

**Table 2:** Autoimmune workup of the patient ANA: antinuclear antibody; anti-dsDNA: anti-double-stranded deoxyribonucleic acid antibodies

Autoimmune workup	Patient value	Reference range
ANA	3+ (nuclear speckled pattern)	
Anti-dsDNA	48 IU/mL	<30 IU/mL
C3 complement	18.23 mg/dL	80–180 mg/dL
C4 complement	<4 mg/dL	10–45 mg/dL

After a few days, the patient developed an acute-onset progressive ascending quadriparesis. Cerebrospinal fluid revealed an albumin-cytological dissociation (mild elevation in protein: 61.9 mg/dl), and nerve conduction studies confirmed acute inflammatory demyelinating polyneuropathy (AIDP). AIDP was managed with plasma exchange therapy. Additionally, he exhibited psychiatric symptoms of intermittent unexplained crying, which was managed with quetiapine. After the resolution of the infection, he was managed with pulse steroid and cyclophosphamide. Over a period of four weeks, he became ambulatory without support. On follow-up, he was in remission with no residual neuropsychiatric sequelae and on a low dose of steroids, hydroxychloroquine, and mycophenolate.

**Figure 5:**
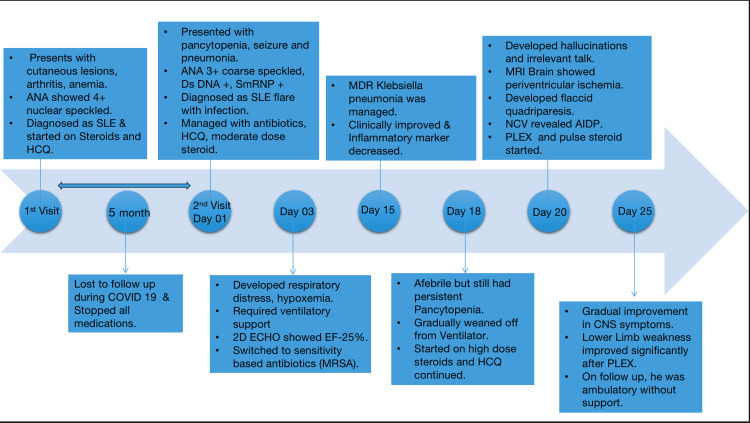
Sequential timeline of various events during the admission period ANA: antinuclear antibody; SLE: systemic lupus erythematosus; HCQ: hydroxychloroquine; dsDNA: double-stranded deoxyribonucleic acid; MDR: multi-drug resistant; MRSA: methicillin-resistant Staphylococcus aureus; MRI: magnetic resonance imaging; NCV: nerve conduction velocity; AIDP: acute inflammatory demyelinating polyneuropathy; PLEX: plasma exchange

A timeline of pertinent events during the admission period of the patient is illustrated in Figure 5.

## Discussion

We presented a patient with an SLE flare in the setting of a coexisting infection. SLE, characterized as "a disease of a thousand faces" [3], is highly heterogeneous, involving diverse manifestations across various body systems. Initially admitted with SLE flare (mucocutaneous and musculoskeletal symptoms), the patient later developed cardiovascular and neuropsychiatric manifestations during the hospital course. The complexity was heightened by superimposed bloodstream and respiratory infections, attributable to compromised immunity. The multifaceted systemic involvement in SLE complicates its management, as infections may overlap with disease activity.

Neuropsychiatric symptoms in lupus exhibit significant variation, with reported prevalence and incidence of 4.3% and 7.8%, respectively [2]. The American College of Rheumatology's consensus statement outlines 19 syndromes categorizing various neuropsychiatric manifestations of SLE as CNS and PNS syndromes, further classifying them as focal or diffuse neurological syndromes [4]. In NP-lupus, the presenting symptoms may include seizure disorder (7-20%), headache (12.2-28.3%), cerebrovascular disease (8-15%), aseptic meningitis (0.3-2.7), psychosis (0.6-11%), and AIDP (0.08-1.2%) [5]. The concurrent presence of CNS with PNS, particularly AIDP, is unusual.

The underlying pathophysiology of SLE is intricate, involving loss of immune tolerance, autoantibody production, immune complex deposition, and faulty clearance of cellular debris. These mechanisms lead to abnormal T and B cell activation, tissue inflammation, and cellular apoptosis [6]. Regarding neurological involvement in lupus, both autoimmune or inflammatory and ischemic or thrombotic pathways contribute to neuropsychiatric manifestations. The autoimmune pathway results in NP manifestations due to inflammatory mediators or autoantibodies, while the ischemic or thrombotic pathway causes cerebral microangiopathy, vascular occlusion, and hemorrhage. Both pathways contribute to the pathology to some extent [7]. Intercurrent infections can initiate a flare and alter the course of illness and outcome. Some infections, like viral Ebstein-Barr virus, cytomegalovirus, gram-negative bacteria, and parasites, play a pivotal role in upregulating SLE activity, while others, like plasmodium and H. pylori, may downregulate inflammation in SLE. Our case involved gram-positive and gram-negative serious infections, posing a significant challenge in countering both the infection and inflammation [8].

No specific diagnostic criteria exist for NP-lupus. Initial steps involve ruling out more common causes such as infections, metabolic issues, intracranial bleeds, and drug-related complications [9]. Diagnosis relies on clinical, serological, immunological, electrophysiological, and neuroimaging studies. However, diagnostic biomarkers in serum and cerebrospinal fluid lack definitive accuracy for clinical use [10,11]. Neuroimaging, even with gold-standard modalities like MRI, may show no significant changes in up to 50% of patients with clinical disease [12]. In our case, clinical, serological, and nerve conduction studies were crucial for diagnosing the neuropsychiatric manifestation, subsequently identified as AIDP.

Managing these cases is challenging, especially when complicated by concurrent infections. Immunosuppressants, including corticosteroids, azathioprine, cyclophosphamide, and mycophenolate mofetil, must be initiated in patients with generalized lupus activity. Among these agents, only prednisolone, antimalarials, and cyclophosphamide have shown evidence of efficacy against neurological symptoms [13]. The use of biological agents, such as rituximab, lacks sufficient clinical data. In our case, the management involved pulse steroids for NP-lupus and plasmapheresis for AIDP. After the resolution of the infection, pulse cyclophosphamide was administered. The question as to whether the infection triggered AIDP remains a challenge for physicians, and there is no diagnostic technique to differentiate between disease flare-ups and infection. Further, larger-scale research is needed to address these questions, enabling the early use of potent immunosuppression. Balancing these considerations has been a decade-long challenge for physicians.

## Conclusions

Neurological manifestations of lupus, particularly AIDP, are infrequent but constitute a devastating condition with significant morbidity. Robust data specific to its management is lacking. Infections may serve as triggers and could mask the symptoms, complicating the decision for potent immunosuppression. Therefore, physicians must exercise caution and use strategic judgment when encountering this challenging condition.
